# Characterization of menstrual stem cells: angiogenic effect, migration and hematopoietic stem cell support in comparison with bone marrow mesenchymal stem cells

**DOI:** 10.1186/s13287-015-0013-5

**Published:** 2015-03-17

**Authors:** Francisca Alcayaga-Miranda, Jimena Cuenca, Patricia Luz-Crawford, Carolina Aguila-Díaz, Ainoa Fernandez, Fernando E Figueroa, Maroun Khoury

**Affiliations:** Laboratory of Nano-Regenerative Medicine, Faculty of Medicine, Universidad de Los Andes, Santiago, Chile; Cells for Cells, Santiago, Chile; Inserm, U844, Montpellier, F-34295 France

## Abstract

**Introduction:**

Stem cells isolated from menstrual fluid (MenSCs) exhibit mesenchymal stem cell (MSCs)-like properties including multi-lineage differentiation capacity. Besides, menstrual fluid has important advantages over other sources for the isolation of MSCs, including ease of access and repeated sampling in a noninvasive manner. Such attributes allow the rapid culture of MenSCs in numbers that are sufficient for therapeutical doses, at lower cell passages.

**Methods:**

In this study, we advance the characterization of MenSC populations in comparison to bone marrow derived mesenchymal stem cells (BM-MSCs) with regards to proliferation, lineage differentiation, migration potential, secretion profile and angiogenic properties *in vitro* and in a matrigel plug assay in mice. We additionally tested their ability to support hematopoietic stem cell (HSC) expansion *in vitro*.

**Results:**

The phenotypic analysis of MenSCs revealed a profile largely similar to the BM-MSCs with the exception of a higher expression of the adhesion molecule CD49a (alpha1-integrin). Furthermore, the fibroblast colony forming units (CFU-F) from MenSCs yielded a 2 to 4 fold higher frequency of progenitors and their *in vitro* migration capacity was superior to BM-MSCs. In addition, MenSCs evidenced a superior paracrine response to hypoxic conditions as evidenced by the secretion of vascular endothelial growth factor and basic fibroblast growth factor and also improved angiogenic effect of conditioned media on endothelial cells. Furthermore, MenSCs were able to induce angiogenesis in a matrigel plug assay *in vivo*. Thus, an 8-fold increase in hemoglobin content was observed in implanted plugs containing MenSCs compared to BM-MSCs. Finally, we demonstrated, for the first time, the capacity of MenSCs to support the *ex-vivo* expansion of HSCs, since higher expansion rates of the CD34 + CD133+ population as well as higher numbers of early progenitor (CFU-GEMM) colonies were observed in comparison to the BM source.

**Conclusions:**

We present evidence showing superiority of MenSCs with respect to several functional aspects, in comparison with BM-MSCs. However, the impact of such properties in their use as adult-derived stem cells for regenerative3 medicine remains to be clarified.

**Electronic supplementary material:**

The online version of this article (doi:10.1186/s13287-015-0013-5) contains supplementary material, which is available to authorized users.

## Introduction

Mesenchymal stem cells (MSCs) are self-renewing progenitor cells with the capacity to differentiate into various cell types under specific conditions. Adult stem cells derived from different sources, including bone marrow, adipose tissue or post-natal tissues, such as umbilical cord and placenta, have been shown to possess regenerative, anti-inflammatory or immunoregulatory potential in a variety of diseases. The limitation of their clinical use resides in the invasiveness of the extraction methods and in some cases their limited proliferative capacity. Furthermore, diverse MSCs sources are known to display distinct functional properties that might contribute to specific therapeutic effects [1].

A study published in 2007, was the first to identify and characterize a new source of stem cells within menstrual fluid. It showed that menstrual-derived stem cells (MenSCs) are rapidly expanded and differentiated under standard laboratory conditions [2]. There is growing interest in their clinical potential since they display a high proliferation rate, are multipotent and obtainable in a periodic and noninvasive manner, devoid of the biological and ethical issues concerning other stem cell types [2-5]. Recent evidence suggests that MenSCs are positive for several MSCs markers, including CD90, CD29, CD105, and CD73, and also remain negative for hematopoietic cell markers, such as CD34, CD45 and CD133. Some reports have demonstrated the expression of embryonic markers and pluripotent intracellular cell markers, such as OCT-4, c-kit and SSEA-4, not found on MSCs from other sources, although these findings have also been disputed, even in cells isolated and cultured under comparable conditions [2-7].

A detailed characterization of the MenSCs is a pre-requisite for head-to-head comparisons with related cell types isolated from other sources, especially the most extensively studied bone marrow derived mesenchymal stem cells (BM-MSCs) that are already in clinical use for specific applications. Since to date there are no ‘potency’ tests available for MSCs, a thorough cell characterization is still a prerequisite prior to the use of a new cell type in clinical applications under safe and effective conditions.

Several studies related to the paracrine angiogenic effects of MSCs have been published since the therapeutic benefits of angiogenesis in different disease models are well-known [8-10]. Meng *et al*. showed that angiogenic factors (ANG-2, vascular endothelial growth factor (VEGF), HGF, and EGF) were constitutively present in the culture media of MenSCs as opposed to cord blood MSCs. The supportive effect of MSCs for hematopoiesis has been well documented in many studies including our published work [11], pointing to the possibility of expanding human hematopoietic stem cells (HSCs), thus increasing their numbers while maintaining their stem cell properties. MenSCs have been tested only in three different animal disease models, exhibiting regenerative properties under different physiological and pathological conditions; however, further investigation is required to substantiate their real therapeutic potential [3-6,12]. From a clinical translational point of view, these stem cells appear to have the robustness to be widely expanded for clinical applications [13] similar to those of BM-MSCs. Indeed, several pilot trials using MenSCs are in the ‘recruitment’ phase (source: clinicaltrial.gov): Subsidiaries of Medistem (AZ, USA) have already allogenically injected 20 patients with heart disease. Furthermore, S-Evans Biosciences Co., Ltd. (China) initiated the recruitment phase of donors for a clinical study to test the effect of autologous MenSCs in liver cirrhosis, acute lung injury and for the treatment of type 1 diabetes in men and women, in this case with allogeneic MenSCs. These trials attest to the fast path stem cell-based therapies are taking during this decade, from the date of their discoveries to the time they reach the clinic.

Aiming at more clarity in this regard, we have characterized several MenSCs derived from multiple donors using a large battery of markers, aiming not only for typical mesenchymal and embryonic markers but also for endothelial and epithelial lineage markers, since these cell types might represent a source of contamination in MenSCs culture. In addition, we have addressed a range of functional properties that contribute to understanding the regenerative potential that could be expected with preclinical or clinical application of MenSCs. These quality-control parameters are essential when comparing similar cells from different sources. We demonstrate that MenSCs have a considerable potential for self-renewal and stable proliferation *in vitro* during a long culture time and a significantly higher migration capacity than BM-MSCs, suggesting they might exhibit several unexpected therapeutic capacities. We also demonstrate that MenSCs secrete higher amounts of angiogenic factors than BM-MSCs, resulting in a higher angiogenic potential both *in vitro* and *in vivo*. Finally, we demonstrate for the first time that MenSCs possess enhanced feeder properties evidenced by their support of HSCs proliferation. The panel of characteristics presented in this article contributes in orienting their clinical use towards the most appropriate applications.

## Methods

### Isolation and culture of cells

All the cells used in this study were harvested with the informed consent of the donor as approved by an Institutional Review Board of Universidad de los Andes and the ethical scientific committee of Clinica Santa Maria and the ‘Servicio de Salud Metropolitano Oriente.’ Menstrual blood-derived stem cells (MenSCs) were collected from five healthy donors ranging in age from 18 to 39 years. Samples were collected in a menstrual silicone cup (Mialuna®, Santiago, Chile) during the earliest days of a menstrual cycle. Menstrual blood samples were transferred into a 50 ml tube with 10 ml phosphate buffered saline (PBS) containing 0.25 mg/ml amphotericin B, penicillin 100 IU, streptomycin 100 mg/ml and 2 mM ethylenediaminetetraacetic acid (EDTA) (all from Gibco, Paisley, UK). Menstrual blood mononuclear cells were separated by Ficoll-Paque Plus (GE Healthcare, Amersham, UK) (1.077 g/ml) density gradient according to the manufacturer’s instructions and washed in PBS. Cells were subsequently cultured in a T25 flask (Falcon®, Becton Dickinson, USA) containing (Dulbecco’s) modified Eagle’s medium (DMEM) high glucose (Gibco) supplemented with 1% penicillin/streptomycin (P/S), 1% amphotericin B, 1% glutamine (Gibco) and 15% fetal bovine serum (FBS) (Lonza, Walkersville, MD USA) at 37°C, 5% CO2 in order to obtain adherent cells. Media were changed the next day to wash non adherent cells. Cells were seeded again as follows: adherent cells were detached using 0.05% trypsin-EDTA (Gibco), counted and sub-cultured. Bone marrow derived mesenchymal stem cells (BM-MSCs) were collected by bone marrow (BM) aspirates from three hip surgery patients (60 to 72 years old). Additional BM aspirates were obtained by iliac crest puncture of a 13 year old patient. BM-MSCs were grown under the same conditions as MenSCs. All the experiments were performed using MenSCs and BM-MSCs from three different donors at early passages (P) P2 to P6. All cells were routinely tested for mycoplasma. HSCs were collected from fresh umbilical cord blood and mononuclear cells were isolated after centrifugation on Ficoll-Paque Plus. HSCs were immunomagnetically isolated using the EasySep™ Human Cord Blood CD34 Positive Selection Kit (Stem Cell Technologies, Vancouver, Canada) according to the manufacturer’s instructions.

### Tri-lineage differentiation

For adipogenesis, cells were seeded at 30,000 cells/well in a four-well plate, in DMEM media supplemented with 10% FBS, P/S and glutamine. When cells reached sub-confluence, the medium was changed to (D)MEM 10% FBS, P/S supplemented with 1 umol/L dexamethasone, 60 umol/L indomethacin and 50 umol/L IBMX for 14 days. Adipocyte differentiation was quantified by measuring the expression levels of peroxysome proliferator-activated receptor (PPAR)-γ by RT-PCR and by the formation of lipid droplets visualized by OIL RED O stain in an inverted light microscope.

For osteogenesis, cells where seeded at 40,000 cells/well in a four-well plate. At confluence, medium was changed to DMEM 10% FBS, supplemented with 0.1 umol/L dexamethasone and 50 ug/mL ascorbic acid in the presence or absence of 3 mM of Na_2_HPO_4_ for matrix mineralization or RT-PCR analysis, respectively, for 21 days. Osteogenesis was quantified by osteocalcin (OC) expression levels by RT-PCR and by Alizarin Red S positive staining.

Chondrogenesis was performed by culturing either BM-MSCs or MenSCs (2.5 × 10^5^ cells) in micropellet in the presence of DMEM supplemented with 0.1 uM dexamethasone, 0.17 mM ascorbic acid, 1% insulin–transferrin–selenic acid (ITS) and 10 ng/ml transforming growth factor b3 (TGFb3) and cultured for 21 days. The chondrogenesis potential was assessed by aggrecan and collagen II RNA expression levels and by positive staining for Safranin O.

### RNA expression

Total RNAs were extracted using the RNeasy kit (Qiagen, Courtaboeuf, France). RNA (0.5 μg) was reverse-transcribed using the M-MLV enzyme (Fisher Scientific, Illkirch, France) and 25 ng of cDNA were amplified using a LightCycler 480 system (Roche Diagnostics, Meylan, France) using the following forward and reverse primers: Aggrecan-forward primer (F): 5′-TCGAGGACAGCGAGGCC-3′, Aggregan-reverse primer (R): 5′- TCGAGGGTGTAGCGTGTAGAGA-3′; Collagen IIA-F: 5′-ACGTGAAAGACTGCCTCAGC-3′, Collagen IIA-R: 5′- AGGAGGTCTTTGGGTCCTA-3′; RSP9-F: 5′-ATGAAGGACGGGATGTTCAC-3′, RSP9-R: 5′- GATTACATCCTGGGCCTGAA-3′; PPARγ-F: 5′-CCAGAAAGCGATTCCTTCAC-3′, PPARγ-R: 5′- TGCAACCACTGGATCTGTTC-3′; Osteocalcin-F: 5′-GGCGCTACCTGTATCAATGG-3′, Osteocalcin-R: 5′- TCAGCCAACTCGTCACAGTC-3′. All values were normalized to either B-globulin or RPS9 housekeeping gene and expressed as relative expression or fold change using the respective formulae 2^− ΔCT^ or 2^− ΔΔCt^.

### Proliferation assay

MenSCs and BM-MSCs were cultured at 1,000 cells/cm^2^ in 24-well plates (Falcon®, Becton Dickinson) in complete DMEM media. Cell proliferation and viability were determined at days 3, 6 and 9 by measuring cellular mitochondrial dehydrogenase using the Quick Cell Proliferation Assay Kit II (BioVision, Milpitas, CA USA) and by spectrophotometric quantification (absorbance, 450 nm) according to the manufacturer’s instructions. Three independent experiments were performed in triplicate. Student’s *t*-test was used to calculate statistical difference. A *P* value ≤0.05 was considered to be significant.

### *In vitro* scratch assay

Cell migration capacity was evaluated in a scratch assay, where cells were grown in six-well plates (Falcon®, Becton Dickinson) to full confluence. A straight scratch of the cell monolayer was performed with a 10 μl pipet tip. Cells were washed with PBS to remove debris and incubated with DMEM 2% FBS for 24 hours. Images were acquired for each sample under a phase-contrast microscope at defined time frames to monitor cell migration into the ruptured area. Migration abilities were quantified by the number of migrated cells inside the scratch area using ImageJ analysis software. The experiment was performed in triplicate. Student’s *t*-test was used to calculate statistical difference. A *P* value ≤0.05 was considered to be statistically significant.

### Colony forming unit assay

To quantify the frequency of stromal progenitors, mononuclear cells obtained after ficoll centrifugation of the menstrual blood were resuspended in DMEM and plated at a density of 100, 1,000, 10,000 and 100,000 nucleated cells/cm^2^. The medium was changed the next day to wash non adherent cells. The frequency of progenitors was calculated following the extreme limiting dilution analysis (ELDA) method for comparing depleted and enriched populations in stem cells [14]. To quantify functional mesenchymal stem cells, MenSCs and BM-MSCs were evaluated for frequency of fibroblast colony-forming units (CFU-F). CFU-F between passage (P) 3 and P 6 were evaluated in a serial dilution assay, where 25 to 250 cells per well were seeded in a six-well plate (Falcon®, Becton Dickinson). After nine to twelve days, cells were stained with 0.5% crystal violet (Sigma-Aldrich, St.Louis, MO, USA) in 10% methanol for 20 minutes. After several washes, colonies formed by more than 50 fibroblast-like cells were counted under a light microscope at low magnification. Results were expressed as CFU/number of cells plated. The Student’s *t*-test was used to detect statistical differences at a *P* value of *P* ≤0.05.

### Immunophenotypic characterization of MenSCs

For cell surface antigen analysis, cells were harvested, washed with cytometer buffer (PBS + 0.2% BSA + 0.01% sodium azide (all from Sigma-Aldrich) and incubated with the specific labelled antibodies in cytometer buffer for 20 minutes at 4°C. Antibodies for human cell surface antigens CD14, CD44, CD90, CD271, CD105, CD73, CD117, CD45, CD34, HLA-ABC, HLA-DR, CD146, EPCAM, CD31, TRA-1-60, CD49a, SSEA3, SSEA4, CD133 and CD3 were purchased from BD Pharmingen™ (BD Biosciences, San Jose, CA, USA), R&D Systems (Minneapolis, MN, USA) and Biolegend (San Diego, CA, USA). In all experiments, matching isotype antibodies were used as negative controls. In addition, LIVE/DEAD®Fixable dead cell stain kit (Invitrogen, CA, USA) was used to determine the viability of cells by flow cytometry according to the manufacturer’s protocol. Data (5,000 events) were collected using a fluorescence-activated cell sorting (FACS) Canto II Flow cytometer (BD Biosciences) and analyzed on Flowjo analysis software.

### Angiogenesis assay

Tube formation in Matrigel was used as a functional assay to evaluate the angiogenic properties of MenSCs. Human umbilical vein endothelial cells (HUVEC) and MSCs were plated at 3,000 cells/cm^2^ and grown until confluence. Cells were incubated for 24 hours in DMEM 2% FBS and then exposed to normoxic and hypoxic conditions (1% O_2_) for 48 hours. Then, conditioned medium (CM) was collected, centrifuged at 500 g for five minutes, and stored in aliquots at −80°C. Cells were trypsinized and resuspended in the CM or in endothelial growth medium (Lonza). Cells were then seeded in triplicate in four-well plates (Falcon®, Becton Dickinson) pre-coated with 200 μl growth factor reduce Matrigel (BD Biosciences, USA) at a density of 6 × 10^4^ cells/well according to the manufacturer’s instructions. After six hours, gels were examined by phase-contrast and three images per well were capture using an Olympus U-RFL-T camera. Covered area, total loops and total length of tube were analyzed using the WinTube software (Wimasis, Munich, Germany). The experiment was performed in triplicate. The Student’s *t*-test was used to calculate statistical difference. A *P* value of *P* ≤0.05 was considered to be statistically significant.

### Quantification of secreted factors by ELISA

MSCs were grown in a six-well plate (5×10^5^ cells/well) and incubated overnight in complete media. MSCs were washed with PBS and cultured for 72 hours with serum-free DMEM in normoxic and hypoxic (1% O_2_,) conditions. Conditioned medium was collected and centrifuged at 500 g for five minutes to remove debris. VEGF and basic fibroblast growth factor (bFGF) levels were detected using VEGF and bFGF duo set ELISA (R&D Systems) according to the manufacturer’s protocol. The Student’s t-test was used to calculate statistical difference. A *P* value of *P* ≤0.05 was considered to be statistically significant.

### *In vitro* expansion of cord blood CD34 + CD133+ hematopoietic stem cells

CD34+ hematopoietic progenitor cells (HPCs) were purified as described previously [11]. The percentage of CD34 + CD133+ HSCs upon enrichment was determined by flow cytometry (76.8% ± 7.99; n = 5). Confluent layers of MenSCs and BM-MSCs were irradiated (15Gy) at the Chilean Commission for Nuclear Energy Facilities. CD34+ hematopoietic progenitor cells were then co-cultured directly in the presence of the feeder layer (contact conditions) or in their absence (HSCs alone) or separated from it by a 0.4 μm microporous membrane (Transwell, BD Bioscience) (noncontact conditions) using StemSpan (StemCell Technologies, Vancouver, BC, Canada) supplemented with human growth factors: 10 ng/ml fibroblast growth factor 1 (FGF-1), 10 ng/ml stem cell factor (SCF), 20 ng/ml thrombopoietin (TPO), 100 ng/ml insulin-like growth factor-binding protein 2 (IGFBP2) (all from R&D Systems, Minneapolis, USA), 100 ug/ml of heparin and P/S (Gibco) as previously described [11]. Briefly, CD34+ HPCs were plated directly on these feeder layers or in transwells above the feeder layer or without stroma in a 1:4 to 1:5 ratio in growth factor-supplemented StemSpan in 24-well plates (Falcon®, Becton Dickinson) at 500 μl/well. Half of the medium was changed every day and was replaced with an equal volume of fresh media.

### Proliferative and phenotypic analysis of CD34 + CD133+ hematopoietic stem cells

All the expanded cord blood cells were harvested after three, five and seven days by vigorous pipetting, washed in PBS, and the total number of live cells determined using trypan blue staining. The fold increase in CD34 + CD133+ HSCs, CD34+ HPCs and total cells was calculated by dividing the number of cells at each day by the initial number of cells plated at day 1. In addition, cells were stained with CD3-APC, CD45-FITC, CD34-PECy7, CD133-PE, and LIVE/DEAD®Fixable dead cell stain. Expression of surface markers was then analyzed by flow cytometry. The absolute number of expanded CD34+ or CD34 + CD133+ cells was obtained by multiplying the percentage of CD34+ or CD34 + CD133+ cells with the total number of cells counted in the same culture well. Three independent experiments were performed each in triplicate. Student’s *t*-test was used to calculate statistical difference. A *P* value of *P* ≤0.05 was considered to be statistically significant.

### Hematopoietic colony assay

Expanded CD34 + CD133+ hematopoietic stem cells were resuspended in Iscove’s MDM plus 2% FBS and plated on methylcellulose-based media (Methocult™ Optimum, StemCell Technologies) according to the manufacturer’s instruction. Cells were plated, in duplicate, at an equivalent final concentration to initial 50 cord blood CD34 + CD133+ hematopoietic stem cells and incubated for 14 days at 37°C with 5% CO_2_ and ≥95% humidity. Burst-forming unit-erythroid (BFU-E), colony-forming unit granulocyte and macrophage (CFU-GM) and colony-forming unit-granulocyte, erythroid, macrophage and megakaryocyte (CFU-GEMM) were classified and counted according to morphological criteria under microscopy. Colonies with more than 50 cells were counted as positive. Student’s *t*-test was used to calculate statistical difference. A *P* value of *P* ≤0.05 was considered to be statistically significant.

### *In vivo* animal models

All *in vivo* studies were performed at the animal facility of the Universidad de los Andes (Santiago, Chile) and received approval by the ‘Universidad de Los Andes ethical committee for animal experimentation.’ In order to evaluate the angiogenic potential of MenSCs, matrigel plug assay was performed in eight-week-old NSG mice (NOD.Cg-Prkdcscid Il2rgtm1Wjl/SzJ, Jackson Laboratories, Bar Harbor, ME, USA). For the matrigel plug assay, mice were randomly divided into four groups (n = 6/8 plugs per group): DMEM (as negative control), HUVEC, BM-MSCs and MenSCs. Growth factor-reduced matrigel (BD Bioscience) was mixed with (D)MEM alone (supplemented with 50 ng/ml VEGF) or 3 × 10^6^ cells (resuspended in DMEM supplemented with 50 ng/ml VEGF), and subcutaneously injected into both flanks of mice. Fourteen days later, matrigel plugs were harvested and processed for hemoglobin quantification. In brief, the matrigel plugs were homogenized in 250 μl Brij-35 solution (Sigma), cleared by centrifugation at 5,000 g for five minutes and the supernatants were collected to measure hemoglobin content with Drabkin’s solution (Sigma) according to the manufacturer’s instruction. Student’s t-test was used to calculate statistical difference. A *P* value of *P* ≤0.05 was considered to be statistically significant.

## Results

### MenSCs exhibit higher proliferation rate, CFU-frequency and migration capacity

**Figure 1:**
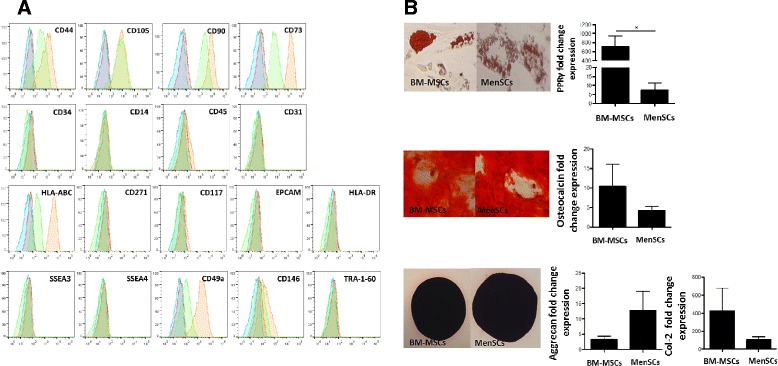
**MenSCs show high expression level of mesodermal antigens and multilineage capacities. (A)** MenSCs display stem cell-like phenotypic markers. In order to determine the immunophenotype MenSCs and BM-MSCs were stained by conjugated antibodies against mesenchymal, hematopoietic and pluripotential stem cells markers, then analyzed by FACS. MenSCs (orange filled histogram) and BM-MSCs (green filled histogram) displayed positive expression for mesenchymal stromal cells while being negative for other markers, such as CD14, CD34, CD45. Besides, low expression for CD146 and high expression for HLA-ABC and CD49a were observed in MenSCs samples. In addition, FACS analysis showed that both cells do not express pluripotent surface markers, such as SSEA-3, SSEA-4, and TRA-1-60. Isotype-matched controls of MenSCs and BM-MSCs are shown with a blue and red filled histogram, respectively. **B)** Differentiation potential of BM-MSCs and MenSCs. Adipose differentiation was characterized according to the fold increased level of the peroxysome proliferator-activated receptor (PPAR)-γ and by the formation of lipid droplets that are positive for Oil red O staining. For osteogenesis characterization, the fold increase level of osteocalcin (OC) and positive staining for alizarin red were evaluated. Chondrogenesis was tested by the expression of collagen IIA (col 2A) and aggrecan (Agg) and by positive staining for Safranin O. Values are expressed as mean ± SE of triplicates (*P ≤0.05). Data shown are representative of multiple replicates. BM-MSCs, bone marrow-derived mesenchymal stem cells; FACS, fluorescence-activated cell sorting; MenSCs, menstrual-drived stem cells; SE, standard error.

Multiple *in vitro* assays were performed to compare distinct properties of MSCs isolated from the menstrual fluid with the gold-standard BM-MSCs. Following cell isolation, MenSCs exhibited spindle fibroblast-like morphology in culture and expressed mesenchymal stem cells surface markers, such as CD105, CD90, CD73, and CD44, in the absence of hematopoietic cell surface markers including CD34, CD45, and CD14. Furthermore, MenSCs lacked the expression of HLA-DR, CD271, CD117, and the endothelial and epithelial surface marker CD31 and EPCAM, respectively. Minimal levels of expression were detected for CD146. While both MSCs sources express HLA-ABC, MenSCs showed higher mean fluorescence intensity. The only prominent immuno-phenotypic difference noted between the two cell sources was the high expression of the α1β1 integrin (CD49a). In addition, MenSCs were negative for the pluripotential surface markers hTRA-1-60, SSEA3 and SSEA4 (Figure 1A). The phenotype identified was stable throughout the subculture period from early passages (P3 to P6) to late passages (P12 to P14) (see Additional file 1: Figure S1).

To evaluate the differentiation capacity of MenSCs into mesodermal tissues, we induced BM-MSCs and MenSCs to differentiate into adipocytes, osteoblasts and chondrocytes. Our results showed that both BM-MSCs and MenSCs were able to differentiate into all three lineages (Figure 1B). However, the quantification of PPRγ levels and oil red O staining revealed that BM-MSCs exhibit significantly higher adipogenic differentiation potential. Nevertheless, no difference was noted when evaluating their osteogenic and chondrogenic differentiation potential by OC and Alizarin red staining and aggrecan, Col2A expression levels and Safranin O staining, respectively.

**Figure 2:**
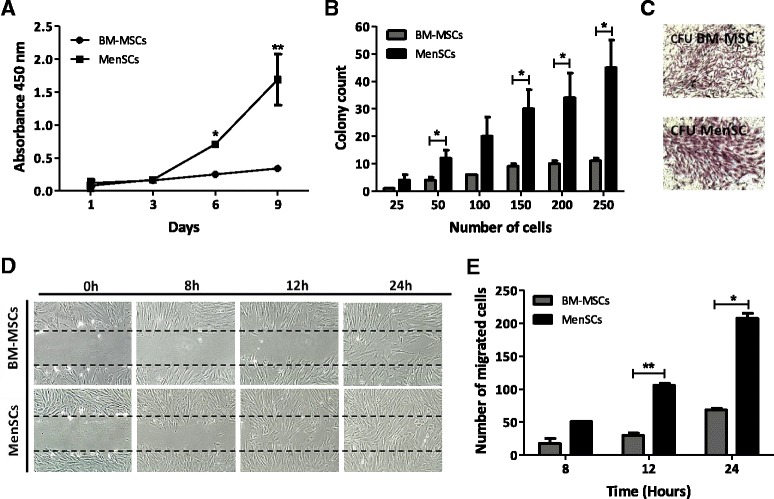
**MenSCs display high proliferation potential, CFU-F capacity, and migration ability. (A)** MenSCs show high proliferation. Proliferation was evaluated using cellular mitochondrial dehydrogenase quantification at different time points. MenSCs showed significantly higher proliferation kinetics with respect to BM-MSCs. **(B)** CFU-F capacity. Serial dilutions of a defined number of cells were cultured and the potential to form CFU was evaluated. MenSCs significantly generated more CFU than BM-MSCs. **(C)** CFU-F morphology of mesenchymal stem cells. At 21 days of culture, the CFU were stained with crystal violet to visualize the colonies generated. **(D-E)** Scratch assay. Confluent monolayers of MenSCs and BM-MSCs were mechanically disrupted with a sterile p10 pipet tip. Images were acquired under a phase-contrast microscope. **(D)** Panels show representative images of BM-MSCs and MenSCs migration post-scratch assay obtained at different time points (magnification 10x). **(E)** Graph represents statistical analysis of scratch assay. Values are expressed as mean ± SE (*P ≤0.05; **P ≤0.01). Data shown are representative of multiple replicates. BM-MSCs, bone marrow-derived mesenchymal stem cells; CFU-F, fibroblast colony-forming unit; MenSCs, menstrual-drived stem cells; SE, standard error.

In accordance with other reports, we observed that MenSCs exhibit a higher proliferation rate [5,7]. The proliferation levels evaluated at culture days 3, 6 and 9 by cellular mitochondrial dehydrogenase measurement, showed a significant 2.84 fold increase (*P* ≤0.05) at day 6 and 4.97 fold increase at day 9 (*P* ≤0.01) of the MenSCs in comparison with the BM-MSCs (Figure 2A). Moreover, the proliferation levels remained unchanged when the cells were cultured for a longer period (P12 to P14) (see Additional file 2: Figure S2).

To evaluate the initial frequency of mesenchymal progenitors in menstrual fluids, the CFU-F assay was performed immediately after ficoll isolation from freshly obtained samples. The frequency of CFU-F was calculated based on the culture of cells isolated from 10 independent samples and serially diluted at numbers ranging from 100 to 10^5^. The value of 1/(CFU frequency) calculated from the limiting dilution analysis was 1 in 10,104 with 95% confidence interval (CI) of 1/4,787 to 1/21,325 (see Additional file 3: Figure S3). Additionally, we performed a CFU-F assay after cell expansion at early passages (P3 to P6) to compare their CFU potential with BM-MSCs. After serial dilution of a normalized number of total cells from both sources, MenSCs generated a significantly higher number of CFUs in comparison to BM-MSCs, with an average 3.5-fold increase in almost all dilutions examined (Figure 2B). The morphology of the colonies generated from the MenSCs was more dense and larger in size and cell number as shown in Figure 2C. In order to assess the stability of their CFU potential, MenSCs were also seeded at a later passage (P12 to P14), with no significant changes noted between early and late passages (see Additional file 4: Figure S4). Furthermore, it is important to note that similar to a previous report regarding donor-to-donor differences for BM-MSCs, MenSCs also presented variability in their properties that were donor dependent [21]. This was evidenced by the comparison of ten samples from different donors in their CFU-potential and absolute progenitor numbers (see Additional file 5: Figure S5).

A scratch wound assay was then performed to examine the migration ability of MenSCs *in vitro* during wound closure. Images taken at different time-lapses post-scratch, showed higher migration potential of MenSCs with respect to BM-MSCs, where complete wound closure was observed within 24 hours. In contrast, BM-MSCs showed only a partial closure after 24 hours (Figure 2D). The number of migrated cells was quantified using digital image analysis software and resulted in a statistically significant higher migratory capacity of MenSCs at 12 hours (*P* ≤0.01) and 24 hours post-scratch (*P* ≤0.05) (Figure 2E).

### MenSCs show higher angiogenic effect on endothelial cells both *in vitro* and *in vivo*

**Figure 3:**
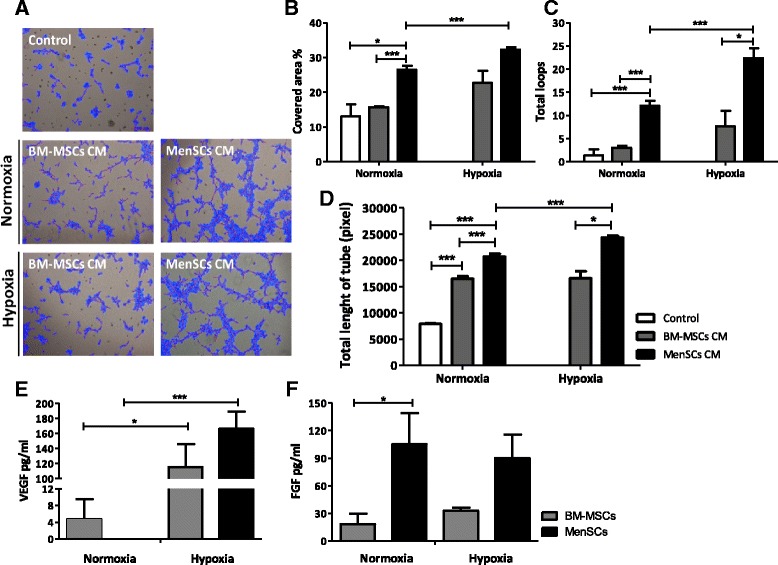
**Conditioned MenSCs media display significantly higher stimulation of tubule structures formation.** HUVEC cells were incubated in MenSCs CM and BM-MSCs CM previously exposed to normoxia (95% O_2_)/hypoxic conditions (1% O_2_) or in basal medium, and cultured for six hours with Matrigel. **(A)** Representative images of the HUVEC tube formation. Panel shows representative images of the capillary network formation on Matrigel assay. Analysis revealed an increase of tubule formation in cells cultured with MenSCs CM, both in normoxia or hypoxia. **(B-D)** Tubules network quantification. Graphs represent the statistical analysis of the percentage of covered area **(B)**, total loops **(C)** and total length of tube **(D)** of tubules network on matrigel angiogenesis assay. HUVEC cultured with MenSCs CM show a statistically significantly higher percentage of covered area, loops and length of tube with respect to the other conditions, both in normoxic and hypoxic conditions. Values shown in the graphs are the mean ± SE (*P ≤0.05; ***P ≤0.001). **(E-F)** Analysis of soluble factors secreted by MenSCs *in vitro*. MenSCs and BM-MSCs cells were cultured for 72 hours under normoxic and hypoxic conditions. **E)** VEGF and **F)** FGF levels of MenSCs and BM-MSCs CM were assessed by ELISA. Values are expressed as mean ± SE; *,*P* ≤0.05; ***,*P* ≤0.001 versus normoxia. Data shown are representative of multiple replicates. BM-MSCs, bone marrow-derived mesenchymal stem cells; CM, conditioned media; FGF, fibroblast growth factor; HUVEC, human umbilical vein endothelial cells; MenSCs, menstrual-drived stem cells; SE, standard error; VEGF, vascular endothelial growth factor.

MSCs share different histological properties that are coherent with endothelial cells enabling them to contribute directly to angiogenesis. They are also capable of participating through the regulation of their secretion of pro-angiogenic factors under an atmosphere of low oxygen that closely approximates the ischemic microenvironment. Hence, the ability of the MenSCs to form a tubule network in semi-solid medium was first tested in an *in vitro* angiogenesis assay. MenSCs and BM-MSCs were resuspended in basal medium and seeded on a three-dimensional matrigel culture system. Tube formation starts to appear at six hours post-culture initiation; however, the difference between BM-MSCs and MenSCs was not significant at that time point (data not shown). In a similar assay, angiogenic factors released by MenSCs and BM-MSCs in the respective conditioned media (CM) were evaluated using HUVEC. In order to mimic an ischemic condition *in vitro*, the effect of CM collected under different oxygen conditions (normoxia versus hypoxia) was evaluated on the capillary-like tube formation of HUVEC (Figure 3). Image analysis of the tube formation showed a more extensive network of capillary-like structures in response to MenSCs CM as compared to BM-MSCs CM or unconditioned control medium (Figure 3A). MenSCs CM cultured under hypoxia showed a significant stimulation in capillary-like structure formation than the medium from cells cultured under normoxic conditions (Figure 3B-D). However, BM-MSCs CM cultured under the different O_2_ levels had no significant difference in tube formation. Under normoxic condition, MenSCs CM showed a higher percentage of covered area (26.48 ± 1.19%) versus BM-MSCs CM (15.68 ± 0.24%, *P* ≤0.0001), higher loop numbers (12.09 ± 1.19 ) versus BM-MSCs CM (3.001 ± 0.47, *P* ≤0.0001) and also longer tube length (20,718.64 ± 531.54 pixels ) versus BM-MSCs CM (16,476 ± 454.14 pixels, *P* ≤0.0001). In accordance with the data presented above, MenSCs CM from hypoxic cultures induced significantly more capillary-like formation than the normoxic condition in all the parameters we evaluated: percentage of covered area (*P* ≤0.001), total loop number (*P* ≤0.001) and total tube length (*P* ≤0.0001). Moreover, the difference in all the parameters mentioned above remained significantly higher in the MenSCs CM conditions. The secreted levels of the angiogenic factors VEGF and bFGF were measured in order to explain the difference in the outcome observed between the two different sources and under different oxygen conditions. Under hypoxic conditions, an important increase of the VEGF secretion level was noted in both CM (24 and 174 folds for BM-MSC and MenSCs, respectively), in comparison with normoxic conditions (Figure 3E). On the other hand, MenSCs CM showed significantly higher bFGF levels in comparison with BM-MSCs CM, under both oxygen conditions (Figure 3F).

**Figure 4:**
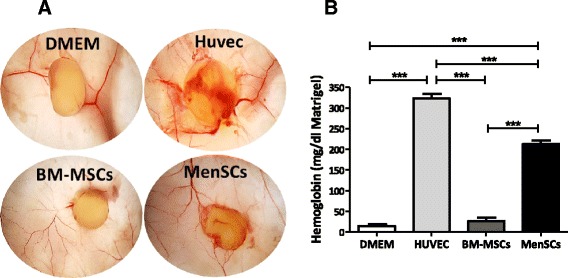
**MenSCs promote angiogenesis** ***in vivo*** **. (A)** Matrigel was mixed with cells (3 × 10^6^ cells/plug) or DMEM alone and subcutaneously injected into mice. After 14 days, the matrigel plugs were harvested and photographed. **(B)** MenSCs have a high hemoglobin content. The hemoglobin content of the plugs from the different groups was measured using Drabkin’s method. The graph represents statistical analysis of the hemoglobin concentration in the matrigel plugs. Values are expressed as mean ± SE (***P ≤0.001). MenSC, menstrual-derived stem cells; SE, standard error.

To evaluate the angiogenic potential of MenSCs *in vivo*, matrigel plug assays were performed in immunocompromised mice. MSCs were mixed with the matrigel and transplanted subcutaneously in mice. At 14 days post-transplantation, plugs were harvested for macroscopic analysis and hemoglobin quantification (Figure 4A). A larger number of blood vessels surrounding the inserted plug was observed with MenSCs; this was correlated with a significant increase in hemoglobin content (212.54 ± 8.72 mg/dl) compared to BM-MSCs (26.5 ± 8.67 mg/dl) or DMEM (14.53 ± 4.95 mg/dl) (*P* ≤0.001), suggesting that pro-angiogenic effects of MenSCs are also seen *in vivo* (Figure 4B).

### MenSCs strongly support the *ex vivo* proliferation of hematopoietic stem cells in a cell contact dependent manner

**Figure 5:**
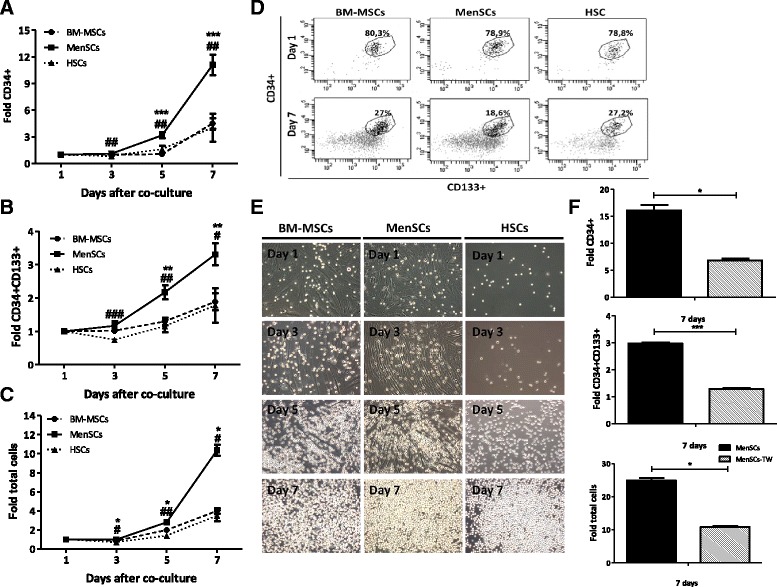
**MenSCs efficiently support the proliferation of CD34+CD133+ hematopoietic stem cells.** Cord blood CD34+ cells were immunomagnetically isolated using CD34 beads, then cultured alone or co-cultured at a ratio of 1:4 to 1:5 in different feeder layers from BM-MSCs and MenSCs (contact conditions) or separated by a transwell (TW) system (non-contact conditions). Total number of cells, the expression of CD34+CD133+ hematopoietic stem cells (HSCs) and CD34+ hematopoietic progenitors cells (HPCs) were evaluated at different time points. **(A)** In vitro expansion of CD34+ hematopoietic progenitor cells. Starting from three days post-culture, a statistically significant difference in favor of HSCs co-cultured with MenSCs was detected. **(B)** In vitro expansion of CD34+CD133+ HSCs. The in vitro expansion of HSCs showed a 3.31-fold increase (±0.33) with respect to the initial cell numbers when they were co-cultured on the MenSCs-feeder layer. **(C)** In vitro expansion of total hematopoietic cell numbers. The graph represents the increase of the total cell number obtained in the co-culture on the MenSCs-feeder compared to the BM-MSCs-feeder or alone. Data represent mean ± SE (*P ≤0.05 compared to BM-MSCs; #P ≤0.05 compared to HSCs). **(D)** Representative FACS analysis seven days post-culture (Dot plot). Percentage of the HSCs at day 1 and day 7. **(E)** Representative morphology of total hematopoietic cells in co-culture with BM-MSCs and MenSCs. The MenSCs-feeder layer showed a higher capacity to increase the proliferation of HSCs compared to BM-MSCs. Original magnification x10. **(F)** Analysis of the effect of non-contact expansion conditions. The ex vivo expansion of CD34+ cells, CD34+CD133+ cells, and total cells decreased under the non-contact condition in comparison with the cell contact cultures. Values are expressed as mean ± SE (*P ≤0.05; ***P ≤0.001). Data shown are representative of multiple replicates. BM-MSCs, bone marrow-derived mesenchymal stem cells; MenSCs, menstrual-drived stem cells; SE, standard error.

MSCs present stromal support properties that can positively impact the expansion and preservation of the ‘stemness’ potential of HSCs/HPCs. In the interest of determining whether the MenSCs possess this property, human umbilical cord blood CD34 + CD133+ cells were isolated and expanded under different culture conditions. First, HSCs were grown directly on confluent MenSCs- or BM-MSCs-feeder layers allowing direct cell-to-cell contact (Figure 5E).

Both co-culture sources were compared to the condition where HSCs were expanded in the absence of feeder cells. Cocultures were maintained for seven days in serum-free defined media containing heparin, SCF, TPO, FGF-1 and IGFBP-2 as previously described [11]. Starting at a density of approximately 1 × 10^4^ cells/well, the expansion of CD34^+^CD133^+^ cells (early progenitors), CD34^+^CD133^−^ (late progenitors) and total expanded cells (CD34^+^ and CD34^−^ cells, presenting differentiated cells) was assessed at different time points of culture (Figure 5). The percentage of expanded hematopoietic progenitors and stem cells was evaluated by flow cytometry, based on the expression of the CD34 and CD133 surface markers at day 1 and 7 of culture (Figure 5D). The proliferation of HSCs *in vitro* occurs both through self-renewal and also differentiation toward early and late hematopoietic progenitors [11]. This explains the decrease in the percentage of the CD34 + CD133+ and CD34+ cells during the expansion time. While the percentage of CD34+ was lower in the condition using the MenSCs-feeder layers, the absolute number of CD34+ cells and, consequently, the level of expansion of these hematopoietic progenitors showed a consistent fold increase of 11.09 ± 1.16. In comparison, the BM-MSCs-feeder layer condition yielded a fold increase of 4.51 ± 0.59 and HSCs alone reached 4.04 ± 1.58. At the end of the experiment, a significant expansion of the CD34+ cells was reached under the MenSCs-feeder layer contact condition with respect to the BM-MSCs-feeder layer (*P* ≤0.0001) or HSCs alone conditions (*P* ≤0.005): 2.46- and 2.75-fold, respectively (Figure 5A). The expansion fold of CD34 + CD133+ hematopoietic stem cells was achieved in the co-culture condition with the MenSCs-feeder layer (3.31 ± 0.33) in comparison with the BM-MSCs-feeder layer (1.89 ± 0.25; *P* ≤ 0.01) or HSCs alone (1.78 ± 0.52; *P* ≤0.05) (Figure 5B). Although, the percentage of CD34 + CD133+ cells on MenSCs was not different, the total number of cells (CD34+ and CD34- cells) obtained was also significantly increased by direct contact with the MenSCs-feeder layer in comparison to both the BM-MSCs-feeder layer and the feeder-free condition support. As expected, total cell numbers were consistently higher with MenSCs-feeder layers compared to BM-MSCs-feeders (*P* ≤0.05) or HSCs alone (*P* ≤0.05), reaching a 10.35 ± 0.57 fold increase (Figure 5C). The expansion values obtained on the BM-MSCs-feeder layer and HSCs alone were: 3.98 ± 0.30 and 3.49 ± 0.59, respectively.

Previous reports derived from *ex vivo* co-culture systems using MSCs as a feeder layer suggest that cell-to-cell contact has a significant impact on cell expansion. In order to determine both the effect of the soluble factors released in the culture and MenSCs contact mediated support, direct and indirect co-cultures between HSCs and the MenSCs-feeder layer (contact condition) were evaluated. To this effect, co-cultures were separated by a microporous membrane that prevented cell contact but allowed exchange of secreted factors. Under these conditions, the expansion of CD34+ cells, CD34 + CD133+ cells and total hematopoietic cells was significantly decreased (2.3 fold) in the non-contact condition for CD34+ cells and total cells. CD34 + CD133+ cells were more affected by the absence of cell contact where a 1.29 fold-decrease was observed (Figure 5F). These results suggest that CD34 + CD133+ HSC expansion is consistently dependent on direct cell interactions with the stromal cells, more than HPCs or hematopoietic cells.

### HSCs expanded on MenSC-feeder layers display higher clonogenic potential

**Figure 6:**
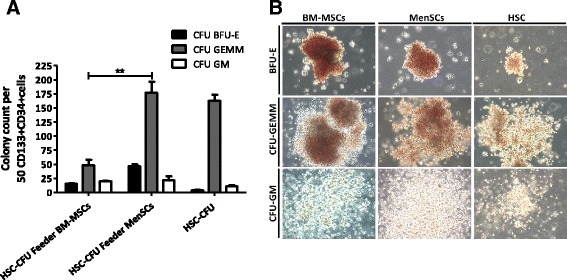
***Ex vivo*** **expansion of CD34 + CD133+ cells on MenSCs-feeder shows high potential of differentiation toward hematopoietic cells.** In order to evaluate the progenitors of the hematopoietic lineage, CFU assay was performed by plating a single cell suspension in a semi-solid MethoCult™ media. At 14 days post-culture, colony types derived from the initial equivalent of 50 cord blood HSC was classified and counted according to morphological criteria. **(A)** Total number of CFU-GEMM, CFU-GM, and BFU-E. Expansion of HSCs on the MenSCs-feeder have a higher capacity to generate CFU-GEMM compared to the other culture conditions. Values are expressed as mean ± SE. (**P ≤0.01). **(B)** Phase-contrast microscopy of representative CFU-GEMM, CFU-GM and BFU-E colonies. Images are representative of each condition. Original magnification x10. Data shown are representative of multiple replicates. BFU-E, burst-forming unit-erythroid; CFU-GEMM, colony-forming unit-granulocyte, erythroid, macrophage and megakaryocyte; CFU-GM, colony-forming unit-granulocyte and macrophage; HSCs, hematopoietic stem cells; SE, standard error.

To assess the changes in the hematopoietic potential of the expanded cells under the different culture conditions, the progeny of 50 CD34 + CD133+ cells was plated on methylcellulose-based media and the number and type of hematopoietic colonies was assessed. Colonies were isolated with the following frequency: for the MenSCs-feeder contact condition the frequency of BFU-E was 30.5 ± 5.5; for CFU-GM, 25 ± 6.0 and for CFU-GEMM, 302.5 ± 17.5; for the BM-MSCs-feeder contact condition the frequency of BFU-E was 24 ± 5.0; and for CFU-GM, 23 ± 6.0 and CFU-GEMM, 97 ± 18. For the HSC alone condition (absence of stroma) the frequency of BFU-E was 1.0 ± 1.0; for CFU-GM, 4 ± 4 and for CFU-GEMM it was 206.5 ± 22.5. As shown in Figure 6A, significant differences (P ≤0.01) were observed in the frequency of the most primitive colonies (CFU-GEMM) originated with MenSCs-feeder support as compared to BM-MSCs-feeders, while no difference was detected in BFU-E and CFU-GM colonies under our three different conditions. Representative images of each type of colony derived from hematopoietic progenitors are shown in Figure 6B.

## Discussion

Menstrual fluids are emerging as an alternative source for the isolation of MSCs, devoid of ethical dilemmas or medical complications for cell harvesting. A major drawback of a single sample harvesting, such as placental and bone marrow sources, is the limited number of culture passages before the cells lose the stem potential deemed crucial for clinical efficacy. The next most important practical feature of MenSCs, besides their accessibility and non-invasive isolation procedure, is the possibility of periodic collections from the same donor. Repetitive collection can ensure higher therapeutic doses of low passaged MSCs from the same genetic background. However, this sole advantage is not sufficient to position menstrual blood as a prominent source for cell therapy. It is essential to contrast these advantages with a broader characterization of the functional properties that might underlie specific therapeutic uses. Multiple efforts have focused on identifying and comparing the cellular and molecular aspects of cell morphology, surface markers, and differentiation capacity of stem cells from the various available sources. Within that scope, we have consequently focused our investigation on looking at different parameters including proliferation and migration rates, and also differentiation, angiogenic and cell feeder potential. For the relevance of the study, all the evaluated measurements were compared head-to-head with bone marrow derived cells, the most broadly used source for MSCs. Both cell sources exhibited MSC features as defined by the International Society for Cellular Therapy (ISCT) minimum criteria [15] including spindle shape, multi-lineage differentiation, and surface marker expression. The phenotypic analysis of MenSCs revealed a profile largely similar to the BM-MSCs with the exception of a higher expression of CD49a (alpha1-integrin subunit). CD49a is an adhesion molecule involved in the regulation of leukocyte migration, proliferation, and cytokine production [16,17]. It has been shown that CD49a enables direct selection and enrichment of human MSCs that express primitive stem cell markers [18]. Also, MSCs derived from embryonic stem cells appear to express a high level of CD49a [19]. Furthermore, another difference in surface marker expression as reported by some groups, was the expression of embryonic markers, such as SSEA-4, in MenSCs that were absent on MSCs from other sources [3,20-22]. However, further results showed a different pattern, where MenSCs isolated and cultured under comparable conditions were negative for the same markers [2]. This raised the question whether these cells represent a more primitive progenitor stage than MSCs from other sources. For this reason, we have tested a larger panel of different embryonic stem (ES) markers including hTRA-1-60, SSEA3 and SSEA4 that were shown to be positive in MSCs isolated from fetal umbilical cords [23]. In our hands, none of these ES markers showed detectable expression on MSCs from the menstrual fluid or BM. Since no epidemiological study is available to date, it is unclear if the discrepancies in the expression of SSEA4, for example, are influenced by age, contraceptive treatments or other environmental factors.

As also demonstrated by others [2,5], the growth kinetics and clonality of MenSCs were significantly higher and these cells could be propagated for longer periods in culture without detectable changes in their proliferation rate. Nevertheless, the proliferation rate is not considered the sole criteria determining a higher abundance of MSCs in one cell source compared with others. The absolute and relative numbers of MSCs and the yield that can be isolated from a single donation are also essential. For this, we have first determined the frequency of CFU-F in menstrual fluid-derived MSCs by serial dilution. Considering the previously estimated number of CFU-F obtained from the bone marrow (about 0.001 to 0.01%), cells isolated from the menstrual fluid yielded a two to four fold higher progenitor frequency, ranging from 0.004% to 0.02%. Considering the average number of mononuclear cells that can be isolated from a single menstrual collection, the estimated absolute number of cells with clonogenic potential is 600 cells/ml (see Additional file 3: Figure S3). To calculate the total number of progenitor cells that can be harvested from the same donor, the number of cells obtained from a single donation should be multiplied by the number of periodic samples obtained from the same donor. The results presented here point to the presence of higher relative and absolute numbers of MSCs in menstrual fluids compared to the bone marrow. It has also been shown that cells displaying a high CFU-F potential are obtained mostly from a CD49a + population [24]. As mentioned previously, MenSCs exhibited a higher expression of CD49a + that could also account for the increased number of CFU-Fs, when similar cell dilution values were used. This attribute is meaningful in relation to their clinical use, since shorter culture times and larger expansion rates can hasten the harvest of the stem cell doses usually required for clinical use (around 1 × 10^6^/kg). The injection of cells at early passages could help avoid reaching the exhaustion of the stem cell properties or senescence that might limit therapeutic effect in patients.

We have also compared the tri-lineage differentiation potential, where MenSCs exhibited a lower adipogenic potential when compared to BM-MSCs. However, it will be interesting to assess more thoroughly the plasticity of these cells in the same comparison scheme, in the differentiation of other lineages. While no such head-to-head comparisons exist, Meng *et al*. showed that these cells can differentiate into nine lineages including cardiomyocytic, neurocytic, myocytic, endothelial, pancreatic, and hepatic [2]. Since cell migration is considered one of the mechanisms behind the MSCs-induced physiological effects in tissue repair, it was essential to understand their capacity to migrate to damaged tissues using an *in vitro* migration assay. MenSCs showed a higher migration potential with respect to BM-MSCs, where a complete wound closure was reached within 24 hours. While the migration capacity of MSCs is under the control of a large range of receptors and chemokines, and their subsequent homing to injured tissues may depend on the systemic and local inflammatory state, further *in vivo* studies are required to fully address this property.

To complete the functional study circle of the MenSCs, their angiogenic potential was assessed *in vitro* using HUVEC culture in normoxic and hypoxic conditions, and *in vivo* in a matrigel plug assay model. We demonstrated that the MenSCs CM was able to induce a significantly higher angiogenic effect on the HUVEC as evaluated by their reorganization and tube formation. We also noted that MenSCs were more responsive to hypoxic conditions, where a statistically significant increase of the secretion of the pro-angiogenic factor, VEGF, was detected. In line with these observations, the MenSCs were more efficient in inducing angiogenesis in the transplanted mice, where an eight fold increase in hemoglobin content was observed in implanted plugs containing MenSCs compared to BM-MSCs.

As MSCs participate in the bone marrow microenvironment by providing growth factors and matrix proteins, it has been demonstrated that they also provide an *ex vivo* support of the expansion of hematopoietic stem cells [11]. Here, we show for the first time, that MenSCs possess strong feeder properties as evidenced by a high expansion, maintenance of stemness and differentiation potential of co-cultured CD34 + CD133+ HSCs. Their support of HSCs cultures was superior to that of the BM-MSCs as a 3X expansion rate of the CD34 + CD133+ population as well as a higher number of early progenitor (CFU-GEMM) colonies was observed. Furthermore, we determined that the stromal support of the proliferation of CD34 + CD133+ cells on both MenSCs and BM-MSCs is cell contact dependent. To claim that MenSCs are the best source for the expansion of HSCs, one should also compare them to other MSC sources used as feeders. Since Klein *et al*. showed that the comparison of MSCs from bone marrow with MSCs from cord blood and amniotic fluid showed no significant difference in the expansion of HSCs [25], one can assume that MenSCs is an important source to consider for the *ex vivo* expansion of HSCs for therapeutic use, where the number of transplanted cells has been an important limitation for the success of the treatment [25]. All the comparative work described here was done using BM-MSC and MenSCs from different donors. It is important to take into consideration donor variation which is a bottleneck for standardization in such studies, as even demographically matched donors can also present different MSC performance as shown in Additional file 5: Figure S5. The ideal situation would be to perform this comparative study using different MSCs sources from the same donors; for this to happen, practical and possible ethical hurdles need to be overcome.

## Conclusions

Whereas a variety of different tissue origins for the isolation of MSCs have been described, the menstrual fluid source may present a large number of advantages. These include being devoid from any ethical controversy, non-invasive and available for their isolation. More importantly, MenSCs possess increased proliferative, migration and pro-angiogenic capacity *in vitro* (especially under hypoxic conditions) and in animal models, in comparison to MSC populations obtained from bone marrow tissues. They also showed a significant stromal cell support for the expansion of HSCs. Therefore, in contrast to the limitations of bone marrow or other one-time harvest sources, such as placental tissues, MenSCs can be obtained repetitively and expanded to the larger quantities required for their therapeutic application. Taking into account all the advantages mentioned above, clinical applications may be based on their regenerative capacity, but more likely on the abundance, frequency, and expansion potential of these cells. However, it remains to determine which specific application is more appropriate for this cell source. It is important to stay away from the presumption that one source of stem cells is superior to other sources in all the existing cell based-therapies. Finally, we present data supporting the MenSCs as a very promising stem cell population. Whether these differences in comparison with BM-MSCs will have significant clinical impact on their functions as adult stem cells for regenerative medicine remains to be investigated.

## Additional files

Additional file 1: Figure S1. MenSCs display a stable expression of different stem markers during long-term culture. To evaluate whether *in vitro* expansion influences the immunophenotype stability of MenSCs, cells were maintained in long-term culture and analyzed comparatively at early (P3 to 6) and late (P12 to 14) culture passages. FACS analysis showed that MenSCs (orange filled histograms) and BM-MSCs (green filled histograms) at early (soft colors) and late (dark colors) culture passages display the same pattern expression of stem cell-like phenotypic markers. Additional file 2: Figure S2. MenSCs show a stable proliferation rate over long-term cultures. In order to evaluate the stability of the MenSCs and BM-MSCs proliferation in long-term cultures, proliferation assays were performed at early (P3 to 6) and late (P12 to 14) culture passages. No statistical differences were observed when comparing cell proliferation at early versus late passages in both MSCs sources. Additional file 3: Figure S3. Limiting dilution analysis of MenSCs defines the frequency of progenitors cells. Mononuclear cells obtained after the ficoll centrifugation of the menstrual blood were resuspended in DMEM and plated at a density of 100, 1,000, 10,000, and 100,000 nucleated cells/cm2. Media were changed the next day to wash non adherent cells. The frequency of progenitors was calculated by counting the number of CFU-F in each determined dilution and following the extreme limiting dilution analysis (ELDA) method for comparing depleted and enriched populations in stem cells. The absolute number of progenitor cells/ml of menstrual blood was obtained by multiplying the frequency of progenitors with the number of total mononuclear cells isolated from 1 ml of menstrual blood post-Ficoll isolation. Additional file 4: Figure S4. MenSCs show a stable colony forming unit potential (CFU-F) in long-term cultures. To evaluate whether long-term expansion affects the MenSCs CFU potential, cells were maintained in culture for multiple passages and analyzed comparatively at early (P3 to 6) and late (P12 to 14) culture passages. Statistical analysis reveals that no significant variation in the CFU potential was observed between early and late passages (left panel). Photographs are representative of the CFU at day 12 (right panel). Additional file 5: Figure S5. MenSCs variability in their properties are donor dependent. Ten samples from different donors were isolated and serial dilutions of a defined number of cells were cultured and their potential for the formation of CFU was evaluated. (A) Total number of CFU colony; (B) Representative images of CFU colony.

## Abbreviations

bFGF: basic fibroblast growth factor
BFU-E: burst-forming unit-erythroid
BM: bone marrow
BM-MSCs: bone marrow derived mesenchymal stem cells
BSA: bovine serum albumin
CFU-F: fibroblast colony-forming units
CFU-GEMM: colony-forming unit-granulocyte, erythroid, macrophage and megakaryocyte
CFU-GM: colony-forming unit-granulocyte and macrophage
CM: conditioned media
DMEM: (Dulbecco’s) modified Eagle’s medium
ELDA: extreme limiting dilution analysis
ELISA: enzyme-linked immunosorbent assay
FACS: fluorescence-activated cell sorting
HPCs: hematopoietic progenitor cells
HSCs: hematopoietic stem cells
HUVEC: human umbilical vein endothelial cells
MenSCs: menstrual derived stem cells
MSCs: mesenchymal stem cells
NSG: Nod scid gamma mice
OC: osteocalcin
PBS: phosphate-buffered saline
P/S: penicillin/streptomycin
TW: transwell
VEGF: vascular endothelial growth factor
