# Supplementary material for: Characterization of menstrual stem cells: angiogenic effect, migration and hematopoietic stem cell support in comparison with bone marrow mesenchymal stem cells
Source: Stem Cell Res Ther. 2015 Mar 17;6(1):32. doi: 10.1186/s13287-015-0013-5 (PMC4404686; doi:10.1186/s13287-015-0013-5)
Supplement: Additional file 1: Figure S1. — MenSCs display a stable expression of different stem markers during long-term culture. To evaluate whether in vitro expansion influences the immunophenotype stability of MenSCs, cells were maintained in long-term culture and analyzed comparatively at early (P3 to 6) and late (P12 to 14) culture passages. FACS analysis showed that MenSCs (orange filled histograms) and BM-MSCs (green filled histograms) at early (soft colors) and late (dark colors) culture passages display the same pattern expression of stem cell-like phenotypic markers. [file 13287_2015_13_MOESM1_ESM.pdf]

Additional File 1

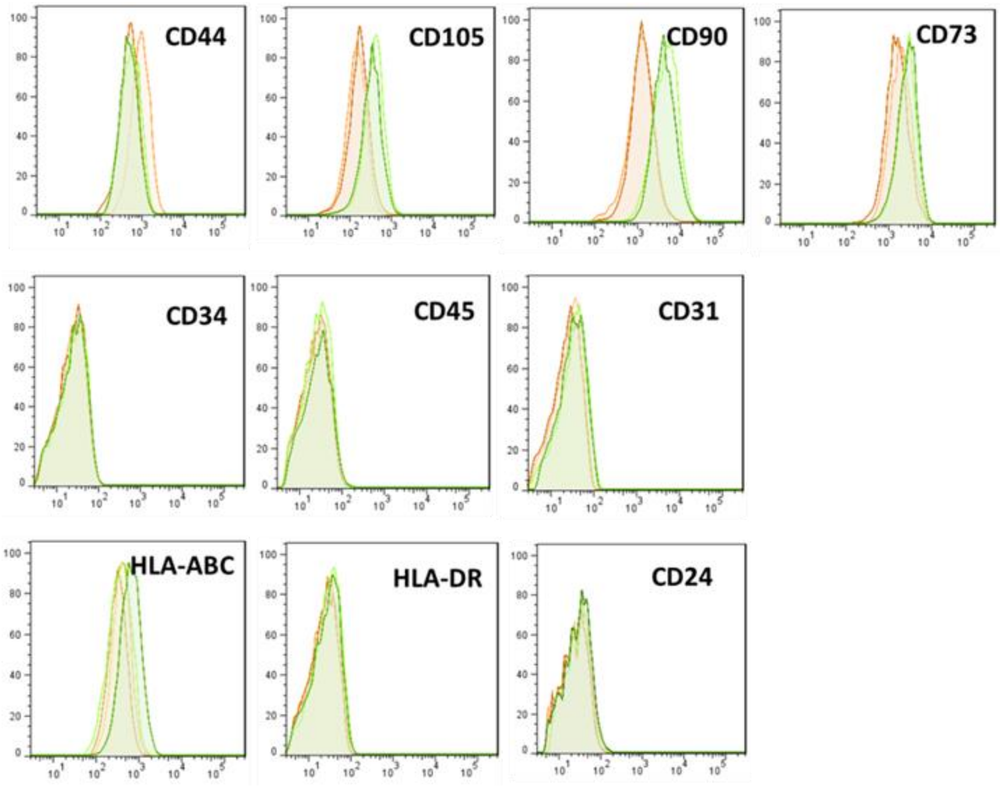

Figure S1. MenSCs display a stable expression of different stem markers during long-term culture. To evaluate whether in vitro expansion influence the immunophenotype stability of MenSCs, cells were maintained in long-term culture and analyzed comparatively at early (P3-6) and late (P12-14) culture passages. FACS analysis showed that MenSCs (orange filled histograms) and BM-MSCs (green filled histograms) at early (soft colors) and late (dark colors) culture passages display the same pattern expression of stem cells-like phenotypic markers.
