# Supplementary material for: Characterization of menstrual stem cells: angiogenic effect, migration and hematopoietic stem cell support in comparison with bone marrow mesenchymal stem cells
Source: Stem Cell Res Ther. 2015 Mar 17;6(1):32. doi: 10.1186/s13287-015-0013-5 (PMC4404686; doi:10.1186/s13287-015-0013-5)
Supplement: Additional file 2: Figure S2. — MenSCs show a stable proliferation rate over long-term cultures. In order to evaluate the stability of the MenSCs and BM-MSCs proliferation in long-term cultures, proliferation assays were performed at early (P3 to 6) and late (P12 to 14) culture passages. No statistical differences were observed when comparing cell proliferation at early versus late passages in both MSCs sources. [file 13287_2015_13_MOESM2_ESM.pdf]

Additional File 2

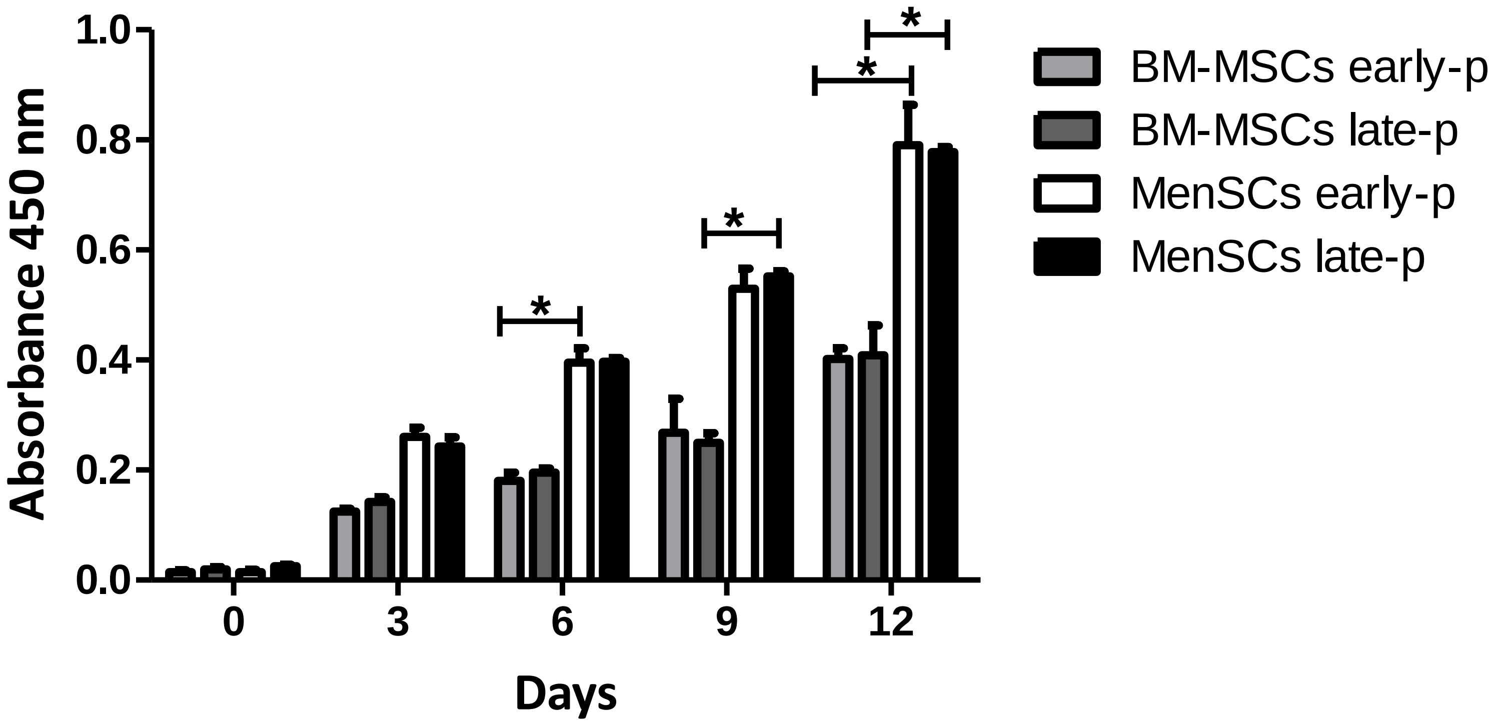

Figure S2. MenSCs show a stable proliferation rate over long-term cultures. In order to evaluate the stability of the MenSCs and BM-MSCs proliferation in long-term cultures, proliferation assays were performed at early (P3-6) and late (P12-14) culture passages. No statistical differences were observed when comparing cell proliferation at early versus late passages in both MSCs sources.
