# Supplementary material for: Characterization of menstrual stem cells: angiogenic effect, migration and hematopoietic stem cell support in comparison with bone marrow mesenchymal stem cells
Source: Stem Cell Res Ther. 2015 Mar 17;6(1):32. doi: 10.1186/s13287-015-0013-5 (PMC4404686; doi:10.1186/s13287-015-0013-5)
Supplement: Additional file 3: Figure S3. — Limiting dilution analysis of MenSCs defines the frequency of progenitors cells. Mononuclear cells obtained after the ficoll centrifugation of the menstrual blood were resuspended in DMEM and plated at a density of 100, 1,000, 10,000, and 100,000 nucleated cells/cm2. Media were changed the next day to wash non adherent cells. The frequency of progenitors was calculated by counting the number of CFU-F in each determined dilution and following the extreme limiting dilution analysis (ELDA) method for comparing depleted and enriched populations in stem cells. The absolute number of progenitor cells/ml of menstrual blood was obtained by multiplying the frequency of progenitors with the number of total mononuclear cells isolated from 1 ml of menstrual blood post-Ficoll isolation. [file 13287_2015_13_MOESM3_ESM.pdf]

Additional File 3

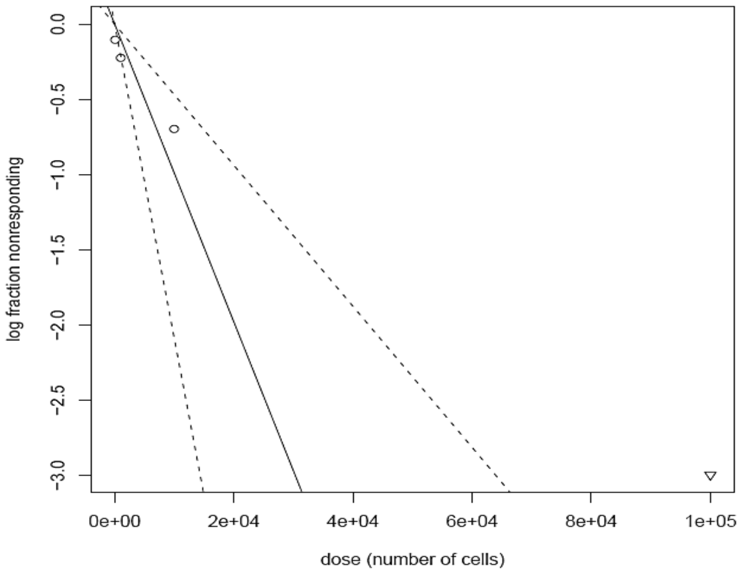

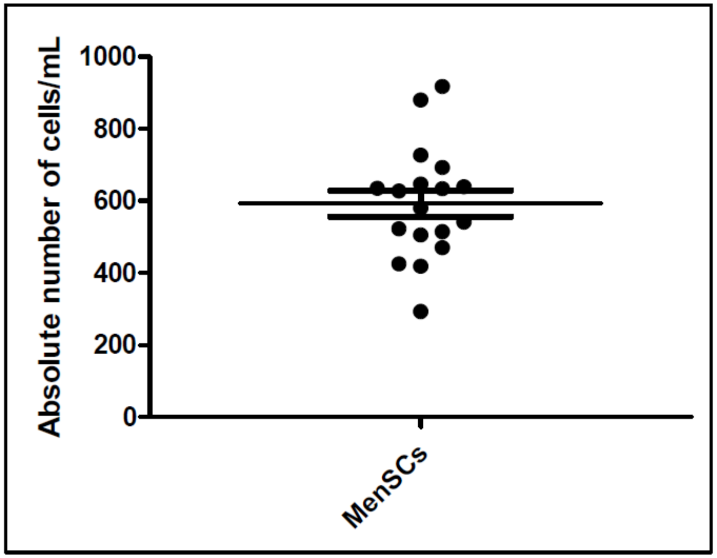

Figure S3. Limiting dilution analysis of MenSCs define the frequency of progenitors cells. Mononuclear cells obtained after the ficoll centrifugation of the menstrual blood were resuspended in DMEM and plated at a density of 100; 1,000; 10,000, and 100,000 nucleated cells/cm^2. Media were changed the next day to wash non adherent cells. The frequency of progenitors was calculated by counting the number of CFU-F in each determined dilution and following the Extreme limiting dilution analysis (ELDA) method for comparing depleted and enriched populations in stem cells. The absolute number of progenitor cells/ml of menstrual blood was obtained by multiplying the frequency of progenitors with the number of total of mononuclear cells isolated from 1 ml of menstrual blood post-Ficoll isolation.
