# Supplementary material for: Characterization of menstrual stem cells: angiogenic effect, migration and hematopoietic stem cell support in comparison with bone marrow mesenchymal stem cells
Source: Stem Cell Res Ther. 2015 Mar 17;6(1):32. doi: 10.1186/s13287-015-0013-5 (PMC4404686; doi:10.1186/s13287-015-0013-5)
Supplement: Additional file 4: Figure S4. — MenSCs show a stable colony forming unit potential (CFU-F) in long-term cultures. To evaluate whether long-term expansion affects the MenSCs CFU potential, cells were maintained in culture for multiple passages and analyzed comparatively at early (P3 to 6) and late (P12 to 14) culture passages. Statistical analysis reveals that no significant variation in the CFU potential was observed between early and late passages (left panel). Photographs are representative of the CFU at day 12 (right panel). [file 13287_2015_13_MOESM4_ESM.pdf]

Additional File 4

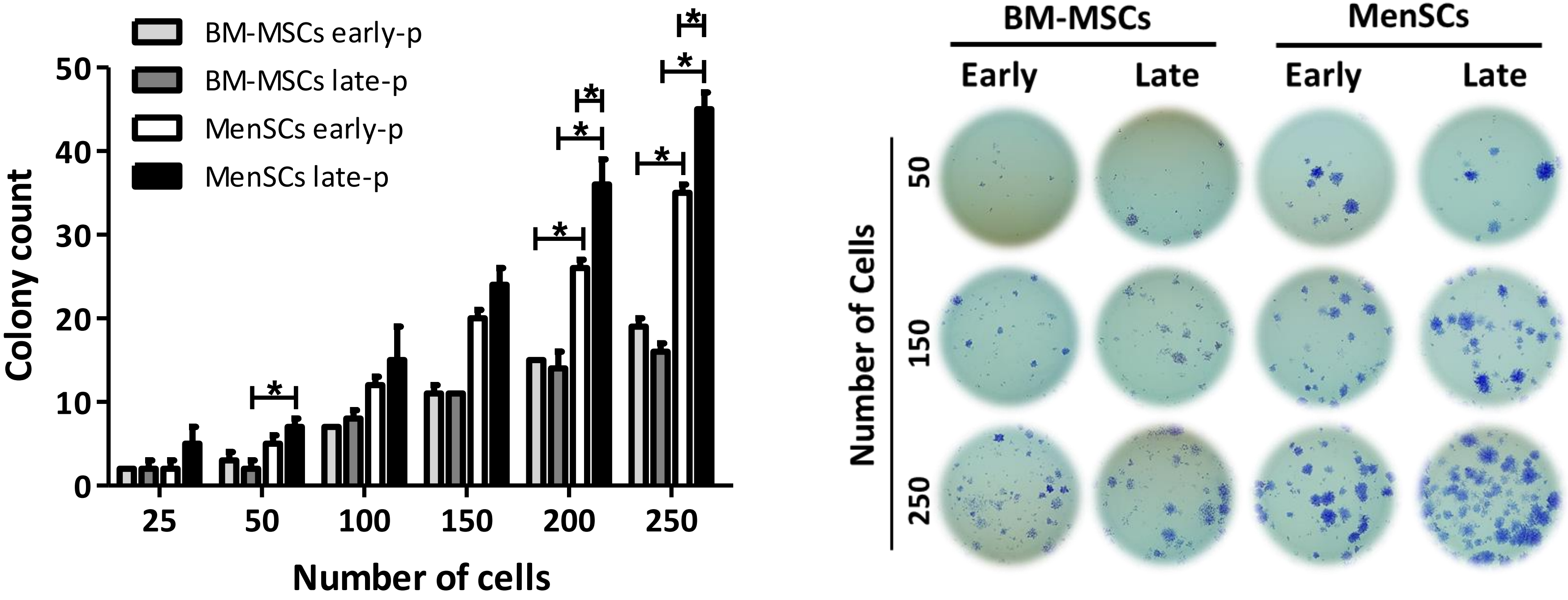

Figure S4. MenSCs show a stable colony forming unit potential (CFU-F) in long-term cultures. To evaluate whether the long-term expansion affects the MenSCs CFU potential, cells were maintained in culture for multiple passages and analyzed comparatively at early (P3-6) and late (P12-14) culture passages. Statistical analysis reveals that no significant variation in the CFU potential was observed between early and late passages (left panel). Photographs are representative of the CFU at day 12 (right panel).
