# Supplementary material for: Characterization of menstrual stem cells: angiogenic effect, migration and hematopoietic stem cell support in comparison with bone marrow mesenchymal stem cells
Source: Stem Cell Res Ther. 2015 Mar 17;6(1):32. doi: 10.1186/s13287-015-0013-5 (PMC4404686; doi:10.1186/s13287-015-0013-5)
Supplement: Additional file 5: Figure S5. — MenSCs variability in their properties are donor dependent. Ten samples from different donors were isolated and serial dilutions of a defined number of cells were cultured and their potential for the formation of CFU was evaluated. (A) Total number of CFU colony; (B) Representative images of CFU colony. [file 13287_2015_13_MOESM5_ESM.pdf]

Additional File 5

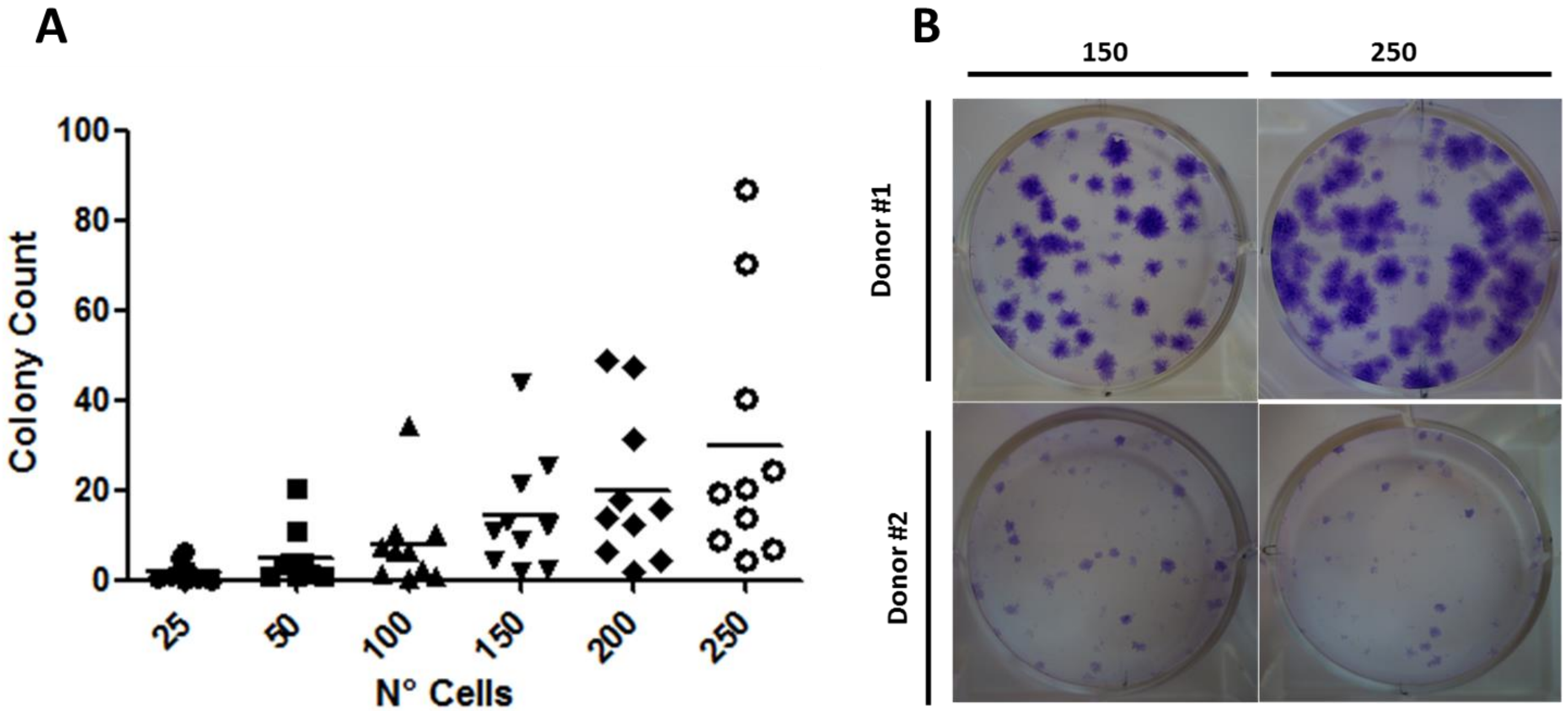

Figure S5. MenSCs present variability in their properties donor dependent. Ten samples from different donors were isolated and serial dilutions of a defined number of cells were cultured and their potential for the formation of CFU was evaluated. **(A) Total number of CFU colony**; **(B) Representative images of CFU colony.**
